# Physiology

**Published:** 1861-05

**Authors:** S. Weir Mitchell

**Affiliations:** Lecturer on Physiology in the Phila. Med. Association


### PHYSIOLOGY.

BY S. WEIR MITCHELL, M.D.,

LECTURER ON PHYSIOLOGY IN THE PHILA. MED. ASSOCIATION.

I.—*Rapidity of the Circulation in the Arteries of the Horse, as indicated by a new Hemodrometer.* By MM. A. CHAUVEAU, G. BERTOLUS, et L. LAROYENNE.

M. Chauveau, dissatisfied with the researches of Volkmann, Huttenheim, Vierordt, Bernard, etc., has constructed a novel form of instrument, designed to exhibit to the eye the rate and power of the arterial current. M. Chauveau accidentally observed that a needle, thrust through the wall of the carotid artery of the horse, moved freely with each passing wave of blood, and again recovered its position under the influence of the elasticity of the arterial coats. To utilize the indications thus offered to the eye, M. Chauveau made use of the following means:—

A short metal tube, of the size of the artery to be examined, was pierced with a hole, over which was stretched a tense caoutchouc membrane. Through this membrane ran a flattened needle, whose exterior extremity traversed a dial-plate attached to the tube. When this tube, thus arranged, was made to replace a part of the artery of a living animal, the moving blood, pressing on the portion of the needle within the tube, caused it to move, and so to register on the dial the extent and character of the forces thus acting within the tube. An opening at the side of the tube permitted the observer to attach to it a manometer, and thus to observe also the pressure of the blood. A series of most interesting experiments was made with this instrument, from which the following conclusions were deduced:—

1. During a single heart-act, (systole, diastole, and pause,) the swiftness with which the blood moves undergoes changes not as yet completely studied.

2. During the heart's systole, the blood of the carotid artery (the vessel chosen for experiment) is put abruptly in motion, with a speed which is relatively considerable.

3. At the end of the ventricular systole, in the moment which immediately precedes the closure of the semilunar valves, the movement of the blood decreases with great rapidity, and may even become altogether null.

4. At the moment when these valves are closed, the circulation experiences a slight acceleration.

5. After the closure of the semilunar valves—that is to say, during the repose of the ventricles—the slight acceleration imparted to the blood current by the dicrotic pulsation, due to the occlusion of the aortic orifice, decreases usually with a certain slowness, since the needle, after this second deviation, does not return suddenly to the zero of equilibrium, or to its neighborhood.

6. At the close of the ventricular diastole the carotid circulation is always inactive, and sometimes appears to be perfectly at repose; the needle at this moment being but slightly, or not at all, deviated from its position of equilibrium.

After pointing out certain facts, which were noticed only in a few of their experiments, the observers relate the means by which they have studied the rapidity of the circulation, and determined that the average rate of speed of the blood in the carotid of a horse is—

During the systole, fifty-two centimetres per second, *i.e.* twenty inches and a half.

During the secondary pulsation, (time, closure of semilunar valves,) twenty-two centimetres, *i.e.* eight inches and seven-tenths.

At the end of the period of repose of the ventricles, fifteen centimetres, *i.e.* five inches and nine-tenths.

In connection with the last valuation, it is proper to add that in some cases the circulation appeared at complete rest during the moment which preceded the ventricular act.

The circulation in a small arterial branch differed from that in the large trunks in the following particulars:—

It was very active, even at the close of the ventricular diastole. The systolic acceleration was relatively much more slight. The acceleration due to the semilunar pulse (la pulsation sigmöide) was feeble, or did not exist at all.

When the glands and muscles of the head, *i.e.* those engaged in mastication, and in salivation, become functionally active, the carotid circulation attained an enormous swiftness, so that five or six times the usual amount of blood was then carried by that vessel.

The authors are of opinion that the condition of the glands when secreting is largely accountable for this curious fact; and they refer to the well-known experiments of M. Bernard, showing that when the submaxillary gland is made to functionate, owing to an irritant having been applied to the chorda tympani nerve, the blood of the veins coming from the gland flows in greatly increased amount.

The influence of hemorrhage, and of division of the pneumogastric and sympathetic nerves, are also the subjects of further experiments. In regard to this latter subject, the authors remarked a notable increase of the rate of circulation in the carotid; and they also observed the same elevation of temperature which M. Bernard and others have perceived. M. Bernard has always held the opinion that this increase of heat was not due alone to the sur-activity of the local circulation, induced by paralysis of the sympathetic nerve, as Dr. Brown-Séquard has contended. M. Chauveau and his companions in the present research seem to incline to the former view, and in support of it urge the fact that the enormous increase in the local circulation which occurs during mastication does not elevate at all the temperature of the face.—(*Journal de la Physiologie*, Oct., 1860.)

### II.—*A Peculiar Form of Deafness.*

At the meeting of the Academy of Sciences on the 8th of January, M. Ménère read a memoir upon "a peculiar form of severe deafness, depending upon a lesion of the internal ear." The subjoined are the conclusions with which he summed up the paper:—

1. An auditory apparatus, up to a certain time perfectly sound, may suddenly become the seat of functional disorders, consisting in noises of a variable character, either continuous or intermittent, and these noises are accompanied by more or less diminution of the sense of hearing.

2. These functional derangements, having their seat in the internal ear, may give rise to complications which may be attributed to cerebral disorders, such as vertigo, bewilderment, uncertain gait, giddiness, and falling; and they may, in addition, be accompanied by nausea, by vomiting, and by a syncopal condition.

3. These complications, which assume an intermittent form, are soon followed by deafness, increasing in intensity; and the hearing is even frequently suddenly and completely extinguished.

4. Everything which has hitherto been ascertained respecting this form of disease leads to the belief that the material lesion which is the cause of these functional derangements is situated in the semicircular canals.—(*London Medical Review*, February.)

The above communication calls forth a note from M. Brown-Séquard on the production of cerebral symptoms as a consequence of lesions of the auditory nerve.

I. I have found that in the batrachia the slightest wound of the auditory nerve suffices to produce the following phenomena:—

1st. A peculiar state of the anterior limb of the opposite side; the member being almost constantly held in contraction, owing to contraction of some of its muscles and paralysis of others.

2d. A marked degree of hyperæsthesia of the skin. These phenomena last during the life of the animal.

Section of the semicircular canals in the batrachia does not produce any of these phenomena unless the auditory nerve has been injured. This nerve, contrary to the views of Müller and Magendie, that some nerves of special sensation are devoid of sensibility, is almost as sensitive in batrachia as the fifth pair.

II. Pricking or section of the auditory nerve in mammals is followed at once by rotatory movements such as follow injuries of the middle peduncle of the cerebellum. The sensibility of the same side of the body is also notably increased by the operation. In the rare cases which survive this operation, a slight paralysis of the side of the body operated on is apt to remain, and rotatory movements are easily reproduced by very slight excitants.

III. Pathological facts observed by R. Bright, by Walter Lincke, Burggrave, and more recently by Hinton, show the influence of lesions of the auditory nerve within the internal ear upon the encephalon. Convulsions, vertigo, and rotatory movements have been observed in cases where the brain was so healthy that it was only possible to regard the phenomena as sympathetic or reflex, and as excited by the lesions of the nerve of hearing.

From these pathological results, from my own experiments, from the effects of a cold injection thrown into the ear, and from the remarkable effects produced by sudden sounds on all nervous or feeble persons, I conclude that the auditory nerve has the power to produce, by reflex agencies, convulsions, vertigo, and other symptoms of disturbed encephalic functions.—(*Gaz. Hebdom.*, Jan. 25, 1861.)

III.—*On the Cerebellum as the Centre of Co-ordination of the Voluntary Movements.* By Jno. C. Dalton, Jr., M.D.

Dr. Dalton has observed, that in a few cases where in birds he had destroyed a large part of the cerebellum, the bird recovered and reacquired the power of co-ordinating its muscular acts. He thus describes an experiment and its results. In the month of January, 1859, I removed from a young but well grown male pigeon, while under the influence of ether, the upper and middle portions of the cerebellum, constituting about two-thirds of its entire mass. Immediately afterward the pigeon showed all the usual effects of the operation, to a very marked degree. He was incapable of walking, of flying, and even of standing still, but struggled and sprawled about, exactly as other pigeons had done when subjected to a similar mutilation. This pigeon, contrary to my expectations, survived, and, in the course of a few days, it was evident that it was recovering the control of its limbs. Its recovery continued to go on at the same time with the general re-establishment of the animal's health; and at the end of five or six days it was again very nearly capable of executing all its natural motions. Its appetite was also restored, and it ate and drank freely as before the operation. The pigeon lived sixteen days, and at the end of that time, when it was killed intentionally by decapitation, there was no apparent reason why it should not have survived indefinitely, since its health, to all appearance, was thoroughly re-established. Its appetite and digestion were good. Its voice was natural in force and tone, and its power of muscular *co-ordination* was so nearly perfect that its deficiency, if any existed, was practically inappreciable. The only peculiarity remaining was a moderate general debility, not noticeable, except when the animal made some unusual exertion. Thus, when he flew off a high perch unto the floor, he found some difficulty in alighting, and generally tumbled forward on his face as he struck the ground, but he always recovered himself immediately and resumed a natural position.

On examination, after death, the wound in the integuments, skull, and membranes, was found to be in rapid process of healing. There was no reproduction of any part of the cerebellum.

Dr. Dalton relates several additional instances analogous to that above recorded, but in which less of the cerebellum was lost. Dr. Dalton thinks that these experiments militate against the views of Flourens, which attribute to the cerebellum the co-ordinating power as regards voluntary muscular movement.—(*Am. Jour. Med. Sci.*, January, 1861, p. 83.)

IV.—*Critical and Experimental Researches on the Function of the Brain.* By R. Wagner.

After destruction of the surface of the cerebellum in pigeons, Wagner observed that there was a tendency to atrophy and absorption of the white matter, which might go on to entire disappearance of the cerebellum, down to the gray nuclei of the cerebral peduncles, (the analogues of the corpora cerebelli.) Small losses of substance, or removal of superficial slices of the cerebellum, with protection of the deep, anterior, posterior, and lateral convolutions, a loss of substance of 70–80 mgrm., often produced no disturbance at all, provided that tearing (zerrungen) was as much as possible avoided. When destruction of the deeper parts was effected, disturbances in movement occurred, which, however, disappeared after a time, ranging from hours to days. Wagner (as also Schiff) believes that this disturbance of the "co-ordination of movement" is not dependent on the cerebellar loss of substance, but upon the tearing of more deeply situated parts, in the performance of the operation. When he succeeded in keeping alive, for weeks or months, pigeons whose cerebellums were completely or in a great measure destroyed, Wagner observed three phenomena, viz., an increasing tendency of the posterior extremities to extension, which was made particularly manifest in a reflectory manner, just as in strychnine poisoning; further, an increasing contortion of the head and neck, so that the latter describes a spiral; lastly, a peculiar quivering of the greatest part of the muscular system, resembling paralysis agitans, and increased on touching the animal. After deep injury of the cerebellum, vomiting was a frequent symptom, also thin, watery fæces. Both of these symptoms also occur in deep injury of other parts of the brain. Digestion is by no means quite suspended after deep injury of the cerebellum, but goes on more slowly and imperfectly. In regard to other general disturbances, of nutrition, of the activity of the skin, heat, etc., Wagner feels doubtful if they depend directly upon the injury of the cerebellum, but holds it as probable that a part of the vaso-motory nerves is represented in that organ. As to the generative system, Wagner could observe no influence of the cerebellar injuries upon it.

In mammals Wagner found confirmed the fact, that mechanical irritations of the cerebellum call forth movements in the organs of the vegetative sphere, in the stomach, intestines, urinary, and sexual organs, also changes in the heart movements. Wagner, from his clinical experience and knowledge of diseases or injuries of the cerebellum, observes that disturbance of equipoise and imperfect staggering motion occur frequently in men with diseased or deficient cerebellums. Further, that rotary movement may also be observed in such cases. Among the most frequent symptoms are paralysis of motion in the extremities, generally in those of the opposite half of the body, or paresis on both sides. It is questionable if these paralytic affections are due to the cerebellar lesion, or are the consequence of pressure on other parts. Paralysis of sensation does not occur, but cramps of the extremities, and epileptic symptoms do; the first in cases of further complication, the last after various injuries of the brain; also general or partial quivering. Vomiting of a periodic character, and symptoms of disturbed digestion, are frequently present. Disturbances of sensibility, pains, particularly in the posterior parts of the head and neck, Wagner recognizes as the most constant symptoms of diseases of the cerebellum. On the whole, he finds a great agreement between the symptoms in man, in other mammalia, and in birds, in regard to the cerebellum, which may, therefore, be considered as physiologically equivalent in warm-blooded animals, without any detriment to the modifications founded upon particular relations of organization.—(*Zeitschr. für Rationelle Medicin; Edinburgh Medical Journal*, January, 1861, p. 234.)

V.—*On Uræmic Intoxication.* By WM. A. HAMMOND, M.D., Professor of Physiology and Anatomy in the University of Maryland.

In the memoir entitled as above, Dr. Hammond relates an elaborate series of experimental researches designed to clear up some of the unsettled questions as to the effects of the accumulation of certain of the urinary elements in the blood. Dr. Hammond considers that the most important of these mooted points is, whether carbonate of ammonia from the decomposing urea, as Frerichs has urged, is the cause of the condition now known as uræmia. After criticising Frerichs's views, by means of a succession of well-planned and ingenious experiments, Dr. Hammond examined the whole subject anew, in an additional series of experiments, and by their aid arrives at the following conclusions:—

1. That the injection of urea, in limited quantity, into the blood of animals produces a certain amount of disturbance in the nervous system, similar in its symptoms to the first stages of uræmia, but that this condition disappears if the kidneys are capable of so depurating the blood as to eliminate the toxic substance.

2. That urea, when introduced into the circulation in larger quantity than can in a limited period be excreted by the kidneys, induces death by uræmia.

3. That by ligature of the renal arteries, or removal of the kidneys, the elements of the urine being retained in the blood, render this fluid unsuitable to the requirements of the organism, and, consequently, induce a condition of system not essentially distinguishable from the uræmic intoxication of Bright's disease, or that caused by the direct introduction of urea into the blood. As, however, was pointed out by Bernard and Barreswil, so long as the urea, or the products of its metamorphosis, are discharged by the stomach or intestines, uræmia does not take place, but that when these channels become closed, convulsions and coma are produced, and death soon follows.

4. That the introduction of urea or urine into the circulation of animals, the kidneys of which have been ablated, shortens the life of such animals, as Frerichs and others have already shown.

5. That there is reason to believe that the urine, as a whole, is more poisonous than a simple solution of urea, for, in those cases in which urine was injected into the blood, the amount of urea thus introduced was much smaller than that previously thrown in, in a pure state, and yet symptoms of as great intensity followed.

6. That urea or the elements of urine, as a whole, induce such a condition of the nervous system as strongly to predispose to congestion and inflammation of the viscera, especially the lungs, pericardium, and spleen.

7. That urea, when directly injected into the blood, or suffered to accumulate in this fluid by extirpation of the kidneys, deranges, in some manner, the process of sanguification, so as to disturb the normal relation of proportion existing between the white and red corpuscles, and either to hasten the decomposition of these latter, or to interfere with the due removal from the blood of such as are broken down and effete.

8. That there is no reason to suppose that, under the circumstances specified, urea undergoes conversion into carbonate of ammonia, but that, on the contrary, there is sufficient evidence to warrant the conclusion that no such process ensues. The fact that in the foregoing experiments a larger amount of urea was generally found in the blood taken from the body after death than in that abstracted during life is, of itself, conclusive against any such hypothesis.—(*Am. Journ. of Med. Sciences*, Jan., 1861.)

### VI.—*Experimental Researches Relative to a Supposed New Species of Upas.* By WILLIAM A. HAMMOND, M.D.

Dr. Hammond gives an interesting history of the two kinds of upas, now known to toxicologists as *Upas antiar* and *Upas tieute.* He quotes the experiments of Leschenault, Magendie, Brodie, etc., to the effect that U. antiar kills by paralyzing the heart, like corroval and vao, the South American arrow poisons, while U. tieute causes the most violent convulsions. In accordance with this, MM. Pelletier and Caventou discovered that the active principle of U. antiar was a new alkaloid, (antiarin,) while U. tieute owed its activity to strychnia. The recent experiments of Prof. Kölliker on U. antiar corroborate the views of the older observers, and discover a striking analogy in the action of U. antiar to corroval and vao.

The upas examined by Dr. Hammond came from Singapore. It was of a dirty-green color, and fæcal odor, and contained strychnia. Upon giving the poison to frogs it caused death with tetanic convulsions: suspecting that some other cause than tetanus was engaged in producing death, Dr. Hammond gave the upas to a frog, and then laid bare the heart. Within five minutes the heart stopped. Meanwhile the frog continued to leap about with unusual vivacity up to the 7th minute, when slight tetanic convulsions took place, and, increasing in violence, caused death at the close of half an hour after the heart stopped.

The upas examined by Dr. Hammond had then the power first to check the heart's acts, and then to cause tetanus. These facts at first led to the idea that it was a mixture of U. tieute and U. antiar, but although it was found to contain strychnia, the other poisonous element which the author obtained from it was not identical with antiarin. This upas cannot, therefore, be looked upon as a mere mixture of those now known.

Dr. Hammond observed that, when taken internally, the action of the two ingredients was reversed as to time, and the tetanus came on first—the heart only ceasing to beat somewhat later. This effect was probably due to a difference in the readiness with which the two poisons were absorbed from the stomach. When, for example, ten drops of a solution of the poison was placed in the stomach, and after a few minutes washed out of the organ, tetanus came on—but the heart continued to beat for several hours.—(*Am. Journ. Med. Sci.*, Oct., 1860, p. 363.)

### VII.—*Schmidt and Stürzwage on the Influence of Arsenious Acid on the Metamorphosis of Matter.*

Schmidt and Stürzwage examined, in common fowls, pigeons, and cats, the influence of arsenious acid on the metamorphosis of matter. They found that small doses of arsenious acid caused a considerable diminution in the excretion of carbonic acid and urea, (from twenty to forty per cent.,) indicating a marked diminution in the metamorphosis of matter. This effect is produced more rapidly when the arsenic is injected into the veins; more slowly, but not less intensely, when it is introduced into the digestive canal. This diminution of the change of matter the authors regard as the cause of the increase of weight in horses treated with small doses of arsenic.—(*Moleschott's Untersuchungen*, vol. vi p. 283; from *British and Foreign Medico-Chirurgical Review*, Jan., 1861, p. 229.)

VIII.—*Zimmerman on Richardson's Hypothesis Regarding the Cause of the Coagulation of the Blood, and some other Views Regarding Fibrin.*

Zimmerman considers Richardson's explanation regarding the cause of the coagulation of the blood as insufficient. Although he does not deny that the volatile ammonia contained in the blood, lymph, and chyle may have some influence on their fluid state, yet their coagulation, he argues, cannot be attributed to the escape of the ammonia, because the blood coagulates in spite of this substance; as, for instance, when blood has been received under mercury and oil, or even after a small quantity of caustic ammonia has been admixed, in which case, although it does not coagulate at once, yet it does so after some hours, when there is still distinct proof that the ammonia has not yet escaped. The theory in question, the author states, is further inadequate to explain the majority of those coagulations which occur in the living body. Zimmerman's own theory is, that the process of coagulation is caused by the formation of a catalytic substance (*contact-körper*) which affects a molecular change in the fluid fibrin, disposing the latter to pass into the solid state. The author cannot give an accurate descripti n of this catalytic substance, but thinks that it exists and remains in a dissolved condition, and that it has only, *in statû nascenti*, the power to act, *per contactum*, on the fibrin.—(*Zeitschr. für Rat. Med.*, vol. viii p. 304, 1860; from the *British and Foreign Medico-Chirurgical Review*, Jan., 1861, p. 229.)

IX.—*Experimental Inquiry into the Composition of some of the Animals fed and slaughtered as Human Food.* By LAWES and GILBERT.

Regarding the *increase* of the weight of animals during the last months of fattening, it is estimated to contain in oxen from 70 to 75 per cent. of total dry substance; of which 60 to 65 parts are fat, 7 to 8 nitrogenous substance, and 1 to 1½ mineral matter. Similar are the proportions in sheep and pigs, but the percentage of fat is larger; that of nitrogenous substance and mineral matter smaller. In many instances the authors have exactly determined the amount and composition of the food consumed during the fattening process, and have compared it with the constituents estimated to be stored up in the increase. Thus they found that in sheep, for 100 of collective dry substance of food consumed, there will be about 9 parts of dry matter in the increase stored up. Of these about 8 parts are fat, and only 0·2 part mineral matter and 0·8 nitrogenous compounds. The average of all estimates relating to pigs showed, for 100 parts of dry matter, food consumed, 17¼ parts of dry increase; of which 18¾ parts were estimated as fat, and rather more than 1⅓ part nitrogenous substance. The authors especially mention the important fact that "there were four or five times as much fat stored up in increase as there was of fatty matter supplied in the food." There was, therefore, obviously a *formation of fat* in the body. On the whole, by the fattening of animals, the proportion of the nitrogenous compounds of the entire body is diminished, and that of fat is increased.—(*Proceedings of the Royal Society*, vol. ix. p. 348, 1859; from the *British and Foreign Medico-Chirurgical Review*, Jan., 1861, p. 225.)

X.—*On the alleged Sugar-forming Function of the Liver.* By PAVY.

Pavy's recent communioation to the Royal Society, on the alleged sugar-forming function of the liver, contains, besides a confirmation of the results of his former experiments, some additional matter. Thus he shows by analysis that although the blood collected from the right side of the heart after death, as was formerly done, affords an abundant indication of the presence of sugar, yet, that when it was removed from the same part by catheterism during life, it is found to contain but a trace of the saccharine principle. Inferences, therefore, having been drawn regarding the ante-mortem state, the author argues, from the post-mortem examinations, must be abandoned as erroneous. Pavy reminds us; at the same time, that slight causes, as disturbance of the respiration, are sufficient to induce a strongly diabetic urine; that therefore a fair specimen of blood from the right heart can only be obtained when the animal is in a quiet state during the performance of catheterism.

Pavy's experiments on rabbits led to the same results as those previously performed on dogs: viz., that injection of starchy and saccharine matter is followed by a great accumulation of hepatine (the sugar-forming substance of Bernard and others) in the liver. While sugar diffuses easily through animal membranes, hepatine has a very low power of diffusion. This opposition strengthens the author in his view that hepatine is not normally transformed into sugar in the living organism. Pavy finally communicates that, after the introduction of large quantities of carbonate of soda into the blood, lesions of the sympathetic system do not cause diabetes.—(*Proceedings of the Roy. Soc.*, vol. x. p. 528, 1860; from the *British and Foreign Medico-Chirurgical Review*, Jan., 1861, p. 231.)

